# IKK2 Inhibition Using TPCA-1-Loaded PLGA Microparticles Attenuates Laser-Induced Choroidal Neovascularization and Macrophage Recruitment

**DOI:** 10.1371/journal.pone.0121185

**Published:** 2015-03-24

**Authors:** Subhash Gaddipati, Qingxian Lu, Ramesh Babu Kasetti, M. Clarke Miller, Qingjun Lu, John O. Trent, Henry J. Kaplan, Qiutang Li

**Affiliations:** 1 Departments of Ophthalmology and Visual Sciences, University of Louisville School of Medicine, Louisville, Kentucky, United States of America; 2 James Graham Brown Cancer Center, University of Louisville School of Medicine, Louisville, Kentucky, United States of America; 3 Department of Chemistry and Biochemistry, University of North Georgia, Oakwood, Georgia, United States of America; 4 Beijing Institute of Ophthalmology, Beijing Tong-Ren Eye Center, Capital Medical University, Beijing, China; 5 Department of Medicine, University of Louisville School of Medicine, Louisville, Kentucky, United States of America; Children's Hospital Boston, UNITED STATES

## Abstract

The inhibition of NF-κB by genetic deletion or pharmacological inhibition of IKK2 significantly reduces laser-induced choroid neovascularization (CNV). To achieve a sustained and controlled intraocular release of a selective and potent IKK2 inhibitor, 2-[(aminocarbonyl)amino]-5-(4-fluorophenyl)-3-thiophenecarboxamide (TPCA-1) (MW: 279.29), we developed a biodegradable poly-lactide-co-glycolide (PLGA) polymer-delivery system to further investigate the anti-neovascularization effects of IKK2 inhibition and *in vivo* biosafety using laser-induced CNV mouse model. The solvent-evaporation method produced spherical TPCA-1-loaded PLGA microparticles characterized with a mean diameter of 2.4 ¼m and loading efficiency of 80%. Retrobulbar administration of the TPCA-1-loaded PLGA microparticles maintained a sustained drug level in the retina during the study period. No detectable TPCA-1 level was observed in the untreated contralateral eye. The anti-CNV effect of retrobulbarly administrated TPCA-1-loaded PLGA microparticles was assessed by retinal fluorescein leakage and isolectin staining methods, showing significantly reduced CNV development on day 7 after laser injury. Macrophage infiltration into the laser lesion was attenuated as assayed by choroid/RPE flat-mount staining with anti-F4/80 antibody. Consistently, laser induced expressions of *Vegfa* and *Ccl2* were inhibited by the TPCA-1-loaded PLGA treatment. This TPCA-1 delivery system did not cause any noticeable cellular or functional toxicity to the treated eyes as evaluated by histology and optokinetic reflex (OKR) tests; and no systemic toxicity was observed. We conclude that retrobulbar injection of the small-molecule IKK2 inhibitor TPCA-1, delivered by biodegradable PLGA microparticles, can achieve a sustained and controllable drug release into choroid/retina and attenuate laser-induced CNV development without causing apparent systemic toxicity. Our results suggest a potential clinical application of TPCA-1 delivered by microparticles in treatment of CNV in the patients with age-related macular degeneration and other retinal neovascularization diseases.

## Introduction

Inflammation in retina is associated with several eye diseases, such as age-related macular degeneration (AMD) [1] and diabetic retinopathy [2]. Prevention of inflammation at early disease stages is sought as a therapeutic approach to avoid irreversible damage to retinal tissue. Inflammation is mediated by a variety of pro-inflammatory cytokines and chemokines. The transcription factor NF-κB controls the expression of a large number of genes under inflammatory conditions [3] and is considered to be an important therapeutic target of different pharmacological agents, including antioxidants and steroid and non-steroid anti-inflammatory drugs [4, 5].

IKK2 is a key upstream kinase necessary for classical NF-κB activation by controlling both the degradation of NF-κB inhibitor proteins and the nuclear localization of the NF-κB dimer [6, 7]. Our recent study showed that the inhibition of IKK2 by genetic deletion or by a pharmacological inhibitor efficiently attenuates laser-induced CNV formation [8] and abnormal choroid blood vessel growth. CNV is a major pathological change associated with wet AMD. The small molecule 2-[(aminocarbonyl)amino]-5-(4-fluorophenyl)-3-thiophenecarboxamide (TPCA-1) is a specific IKK2 inhibitor and can efficiently inhibit NF-κB activation either in culture or in animal models of chronic inflammation [9]. We have shown that TPCA-1 inhibits activation of NF-κB and expression of angiogenic factors in both cultured human ARPE-19 cells and in choroid *in vivo* and is therefore able to attenuate laser-induced CNV formation *in vivo* [8].

IKK2 chemical inhibitors have been widely and intensively investigated as targets for anti-inflammatory and anti-tumor therapies [10–12]. However, systemic toxicity prevents these inhibitors from becoming effective therapeutic drugs [13–16]. Meanwhile, local IKK2/NF-kB inhibition within the eye (periocular or intravitreal) achieves a therapeutic effect while avoiding systemic toxicity [8], suggesting its potential for treating eye diseases. AMD is a chronic disease that needs the therapeutic level of a drug to be maintained for a prolonged period. Frequent intravitreal or periocular injections generate adverse effects and the risk of complications. Therefore, an efficient drug delivery system with sustained and controlled intraocular release is needed. A number of approaches have been explored to achieve this purpose, including coupling the drug to liposomes, microparticles (1–1000 μm), and nanoparticles (1–1000 nm, generally 20–300 nm) [17]. The most commonly used polymers for drug packing are polylactide (PLA), poly-lactide-co-glycolide (PLGA), and acrylic, all of which can be degraded *in vivo* to form natural metabolites [18]. Drug release rates can be regulated by changing polymer chemical composition and molecular weight to achieve long-term delivery, ranging from several weeks to months after a single administration. Both micro- and nanoparticles are currently being evaluated as a potential drug delivery option for AMD patients [19]. The difference in size between micro- and nanoparticles has numerous effects on their properties; most importantly, the larger microparticles have higher maximal drug loading and slower drug release than the smaller nanoparticles [20].

In the present study, we developed a biodegradable PLGA polymer-delivery system to investigate the anti-neovascularization effects of the IKK2 chemical inhibitor TPCA-1 in a mouse model of laser-induced CNV formation. The solvent-evaporation method was used to fabricate TPCA-1-loaded PLGA microparticles for better local and extended effects of TPCA-1, because it produces polymers with higher encapsulation efficiency and more sustained release of therapeutic doses of TPCA-1 *in vivo*. The results show that retrobulbar injection of TPCA-1, delivered by biodegradable PLGA microspheres, can achieve a sustained and controllable drug release into choroid/retina tissues and attenuate laser-induced CNV development and macrophage recruitment without systemic toxicity. Inhibiting NF-κB/IKK2 using PLGA polymer-packed small molecular chemical inhibitors could be a potential therapeutic approach for treating the inflammation and angiogenesis-associated eye diseases.

## Materials and Methods

### Ethics statement

All animal experiments were approved by Institutional Animal Care and Use Committee the Animal Care Committee at the University of Louisville (IACUC #12010) and were conducted in accordance with the guidelines of Association for Research in Vision and Ophthalmology on the use of animals in research.

### Animals

Wild-type C57BL/6J mice (The Jackson Laboratory, USA) between 6 and 8 weeks of age were used and maintained at a maximum of five littermates per cage, with food and water ad libitum, and at constant temperature (20°C±2°C) with a 12 hour light/dark cycle. The wild-type C57BL/6J mice were employed in all the experiments and the number of mice for each set of experiments was used as follow, n = 12 for detection of the TPCA-1 level in posterior eye tissues after TPCA-1-loaded PLGA retrobulbar injection, n = 12 for assessment of retinal histology and visual function, n = 12 for histological studies, and n = 9 for flow cytometric analyses of liver, spleen, and T cell populations, n = 28 for evaluation of the effects of retrobulbar-injected TPCA-1-loaded PLGA microparticles on laser-induced CNV formation, and n = 53 for studies of the effects of TPCA-1-loaded PLGA microparticles on macrophage recruitment to the laser lesions. For all applications, mice were euthanized by CO_2_ exposure followed by cervical dislocation.

### Chemicals

Poly (D,L-lactide-co-glycolide), ester terminated, MW 7,000–17,000 (RESOMER RG502; cat. no. 719889), polyvinyl alcohol (Cat. No. 363170), dichloromethane (Cat. No. 270997), dimethyl sulfoxide (DMSO; cat. no. D2650), and hypromellose (cat. no. H3785) were obtained from Sigma-Aldrich Chemical Co. (St. Louis, MO, USA). The 2-[(Aminocarbonyl)amino]-5-(4-fluorophenyl)-3-thiophenecarboxamide (TPCA-1; cat. no. 2559) was purchased from Tocris Bioscience (Bristol, United Kingdom).

### Fabrication of TPCA-1-loaded PLGA microparticles

TPCA-1-loaded PLGA microparticles were fabricated using a solvent-evaporation method [21, 22]. PLGA (100 mg, 50:50) was briefly dissolved in 1.8 mL of dichloromethane (DCM), and 10 mg of TPCA-1 was dissolved in 200 μL of DMSO. The two solutions were mixed together with a homogenizer for 1 min at 10,000 RPM, and the resulting solution was added slowly to 10 mL of an aqueous polyvinyl alcohol (2% w/v) solution while homogenizing at 10,000 rpm to obtain an oil-in-water emulsion. The contents were stirred (1000 rpm) overnight at room temperature to evaporate the methylene chloride, allowing the formation of a turbid particulate suspension. The microparticles were separated by centrifugation of the suspension at 1000 x g for 30 min at room temperature. The pellet was washed twice with deionized water and lyophilized to allow the microparticles to encapsulate the TPCA-1.

### Quantification of TPCA-1 by HPLC

The TPCA-1 content in the DCM solvent-extracted samples was quantitated with a Waters Alliance e2695 HPLC/Auto-sampler system equipped with a Waters 996 UV-vis photodiode array detector operated by Empower software (Waters) with a μRPC C2/C18 ST 4.6/100 column (GE Healthcare) equilibrated with water/acetonitrile. The flow rate was set to 0.5 mL/min. Initial injection conditions were 100% H_2_0 with 0.1% TFA for 5 minutes, immediately followed by a 30-minute linear gradient to 100% acetonitrile. Acetonitrile (100%) was maintained for 5 minutes before returning to 100% H_2_O/TFA conditions. Spectrophotometric data was collected from 200–800 nm, and the baseline was monitored at 260 nm and 280 nm. An extracted chromatogram at 310 nm was used for calibration purposes. A standard curve was created using the peak area generated by a set of standard solutions ranging from 500 μM to 0.1 μM. Authentic TPCA-1 eluted at approximately 21 minutes. As an additional check to confirm the identity of the peak presumed to be comprised of authentic TPCA-1 fractions of the highest concentration, calibration standards were collected and submitted for mass spec analysis using an Agilent Technologies 6224 TOF-LC/MS system equipped with 1260 series Infinity HPLC/Auto sampler/UV-vis detector and Mass Hunter software. Lyophilized unknown samples were dissolved in acetonitrile, spun down, and aliquots were placed into sample bottles. The samples were then run on the HPLC, and the results were evaluated in comparison with the previous linear calibration.

### TPCA-1 loading efficiency

The loading efficiency of TPCA-1-loaded PLGA microparticles was determined by the extraction and quantification of free relative to encapsulated TPCA-1. PLGA/TPCA-1 microparticles (10 mg PLGA/1 mg TPCA-1) were briefly suspended in 1.5 mL of methylene chloride and incubated at room temperature on a shaker. The resulting solution was mixed with 1.5 mL acetonitrile. This solution was mixed on a vortex for 1 min and centrifuged at 18,000 x *g* for 10 min, and the TPCA-1 levels were then measured by injecting 150 μL of 1:500-diluted supernatant into the HPLC as described above.

### Retrobulbar injection

Mice were anesthetized with an intra-peritoneal injection of ketamine hydrochloride (0.0613 mg/g body weight) and xylazine hydrochloride (0.0245 mg/g body weight). The anesthetized mouse was positioned on its side under a stereo microscope, and the mouse head was restrained with thumb and middle finger. The needle was inserted at 45° to the eye, from the lateral to the medial canthus. The tip of the needle was positioned behind the eye globe, and 100 μl of PLGA microparticle suspension (10 mg PLGA in PBS) or TPCA-1-loaded PLGA microparticle suspension (10 mg PLGA/0.8 mg TPCA-1 in PBS) was gradually injected.

### Detection of the TPCA-1 level in posterior eye tissues after TPCA-1-loaded PLGA retrobulbar injection

Eight-week-old C57BL/6J mice received 100 μl of TPCA-1-loaded PLGA microparticle suspension (10 mg PLGA/0.8 mg TPCA-1 in PBS) in one eye. At 1, 7, 14, or 21 days after injection, both eyes were enucleated and washed at least four times in PBS. The anterior segment, lens, and sclera tissue were removed, and the remaining choroid/RPE/retina complex was retained, homogenized in DCM, and centrifuged at 12,000 RCF/4°C to remove tissue debris. The supernatant was used for quantitation by HPLC as described above.

### Laser treatment

The procedure was performed as previously described [8]. Laser photocoagulation (Elite 532- nm laser, 250 mW, 50 ms, 50 μm; Lumenis Inc., Salt Lake City, UT, mounted on a slit lamp) was briefly bilaterally performed (4 spots/eye) for each animal. The laser spots were aimed around the optic nerve and away from blood vessels. The corneal refractive power was compensated using a plastic coverslip coated with 2.5% hypromellose in saline (Sigma-Aldrich, H3785) [23].

### Optokinetic reflex (OKR) measurements

Visual function was assessed using a non-invasive OptoMotry© optokinetic testing system (CerebralMechanics Inc., Lethbridge, AB, Canada), following the procedures described previously [8].

### Fluorescein retinal angiography

Seven days after laser photocoagulation, fluorescein angiography was performed using a stereo microscope (Zeiss, SteREO Discovery V8 with X-cite light source) as previously described [8]. Photographs were taken after dilation with a single drop of a 0.06% tropicamide and 0.3% phenylephrine hydrochloride mixture and intraperitoneal injection of 50–100 uL of 10% Fluorescein Sodium solution (HUB Pharmaceuticals, CA, USA). Leakage spots were graded as previously described [8]. Evaluation of fundus photographs was done after masking for treated and untreated groups.

### Staining and imaging of CNV

For isolectin IB4 staining, laser-injured eyes were enucleated and fixed with ice-cold 4% paraformaldehyde at 4°C overnight. The anterior segment, lens, and neural retina were removed, and the remaining eye cups with attached RPE layer were washed with 1xPBS and incubated with Alexa Fluor 568-conjugated isolectin GS-IB4 (1 mg/mL, 1:100 in 1xPBS, Invitrogen, Oregon, USA) overnight at room temperature. The eye cups were washed with 1xPBS and flat-mounted with a radial cut. The images were taken under a fluorescence microscope (Carl Zeiss MicroImaging GmbH) immediately after the staining procedure [24].

### Staining and imaging of macrophages

Following enucleation, eyes were immediately fixed in 4% paraformaldehyde at 4°C overnight. Using a dissecting microscopy, the posterior eye cup with attached RPE was separated for macrophage staining of the choroid/RPE, washed with 1xPBS, and incubated with rat anti-F4/80 antibody (ab6640, 1:100, Abcam, Cambridge, MA, USA) overnight at 4°C. The eye cups were washed with 1xPBS and flat-mounted with a radial cut. The images were taken under a fluorescence microscope (Carl Zeiss MicroImaging GmbH) immediately after the staining procedure.

### Measurement of laser-induced CNV area

The area of GS-IB4-stained CNV was measured using AxioVision software (Carl Zeiss MicroImaging GmbH). All data were collected as the total CNV area per eye per mouse and are presented as the relative CNV area compared with control mice. Data was expressed as mean±SD, and statistical analysis was performed using a paired Student’s t-test.

### Histology

Tissues were fixed in 10%-buffered formalin and embedded in paraffin. Serial sections (5 μm thick) of eyes, liver, and spleen were generated. Sections were deparaffinized, rehydrated with distilled water, and stained with hematoxylin and eosin (H&E). Sections were observed under a bright-field microscope.

### Flow cytometry

Flow cytometry was performed as previously described [8, 25]. Briefly, single-cell suspensions were prepared from thymus and spleen of the mice at 7 days after receiving either a single retrobulbar injection of a 100-μl TPCA-1-loaded PLGA microparticle suspension (10 mg PLGA/0.8 mg TPCA-1 in PBS), or a control sham-loaded PLGA microparticle suspension (10 mg PLGA in PBS), or no injection. The cell suspension was then passed through 70-μm nylon mesh (Fisher Scientific). After lysis of RBCs using ACK buffer (0.83% ammonium chloride, 0.1% potassium bicarbonate, 0.037% EDTA [pH 7.2]), Fc receptors were blocked with anti-CD16/32 antibody (Ab) before staining. To stain cell surface antigens, 10^6^ cells in 50 μL of staining buffer (1xPBS containing 3% BSA and 0.1% sodium azide) were incubated at 4°C for 30 min with fluorochrome-conjugated mAbs; to stain apoptotic cells with annexin V, cells were resuspended in an annexin V binding buffer (Biolegend, CA) and incubated with rat anti-TCRbeta and PE-labeled-annexin V reagent (Biolegend, CA). Ab-stained cells were analyzed on a four-color BD FACSCalibur cell analyzer (BD Biosciences), and the data were analyzed with CellQuestPro 5.1.1 software (BD Biosciences). The following antibodies were used: rat anti-mouse CD16/32 blocking Ab (clone 2.4G2) obtained from BD Biosciences (San Diego, CA); rat anti-mouse TCR-β-FITC (H57–597), CD8a-PE (53–6.7), CD4-APC (GK1.5), and PE-conjugated rat IgG2a or IgG2b isotype Ab controls purchased from eBiosciences (San Diego, CA).

### RNA isolation and quantitative (q) PCR

The total RNA from the RPE/Choroid tissues of laser injury eyes were extracted using TRIzol reagent (Invitrogen). The A260/A280 ratio of all RNA samples was >2.0, as measured by Nanodrop. Double-stranded cDNA was reverse-transcribed using random primers and the SuperScript VILO cDNA synthesis kit (Invitrogen).

Real-time qPCR was performed in a SYBR green-based PCR reaction mixture on a MX3005p system (Agilent Technologies, Inc., Santa Clara, CA), programmed with a 10-minute initial hot-start activation of *Taq* polymerase at 95°C, followed by 40 cycles of amplification (95°C for 10 seconds, 56°C for 5 seconds, and 72°C for 10 seconds). The comparative threshold cycle (CT) method normalized to *Gapdh* was used to analyze relative changes in gene expression. The qPCR primer sequences for *Gapdh* have been published elsewhere [26]. The primers for mouse *Vegfa* (ID 6678563a1) and*Ccl2* (ID 141803162c1) were designed based on the online PrimerBank database (Harvard Medical School, Boston, MA; http://pga.mgh.harvard.edu/primerbank).

## Results

### Preparation of PLGA microspheres and characterization of particle size

**Fig 1:**
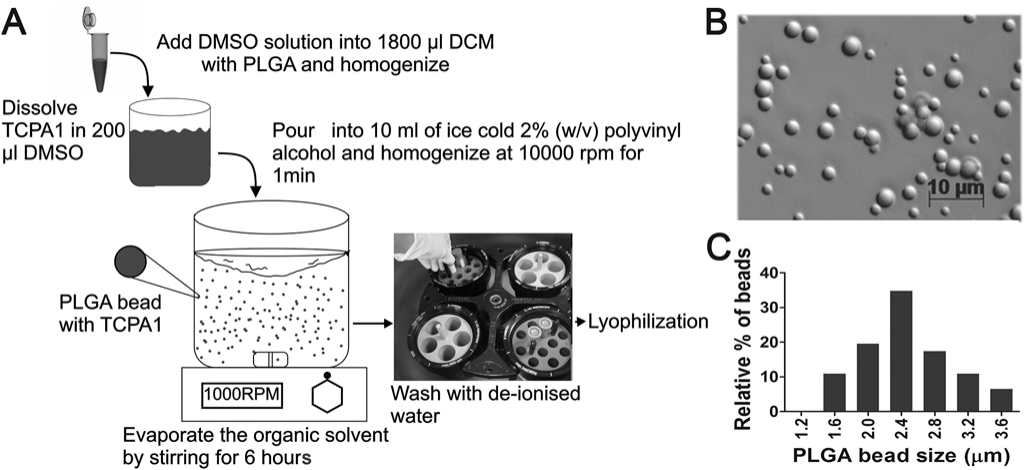
Preparation of PLGA microspheres. (A) Schematic for preparation of TPCA-1/PLGA microspheres. (B) Microscopic images of microparticles loaded with TPCA-1. Scale bar, 10 μm. (C) Size distribution of TPCA-1*/PLGA microparticles*. The mean diameter is 2.4 μm.

TPCA-1 is a water-insoluble small molecule and can be loaded into PLGA microparticles by a single-emulsion solvent-evaporation method [21, 27, 28]. A TPCA-1-loaded PLGA polymer fabrication procedure was developed, as illustrated in Fig. 1A. One hundred mg of PLGA (MW 7,000–17,000, lactide:glycolide [50:50]) was dissolved in 1.8 ml of dichloromethane prior to the addition of 10 mg of TPCA-1 dissolved in 200 μl DMSO. This polymer solution was homogenized at 10,000 rpm for 1 min. The mixture was then poured into 10 ml of a 2% w/v polyvinyl alcohol (PVA) aqueous solution and emulsified with a high-speed homogenizer at 10,000 rpm for 1 min. The organic solvent was allowed to evaporate by stirring the mixture at 700 rpm for 18 hours under ambient temperature. PLGA microparticles were characterized by their size distribution determined using a Zeiss Axio Imaging system with a 60x oil objective lens. Following this procedure, *homogeneous* TPCA-1-loaded PLGA *microparticles were generated* with an average diameter of ~2.4 μm (Fig. 1B, C).

### Determination of drug-encapsulation efficiency

**Fig 2:**
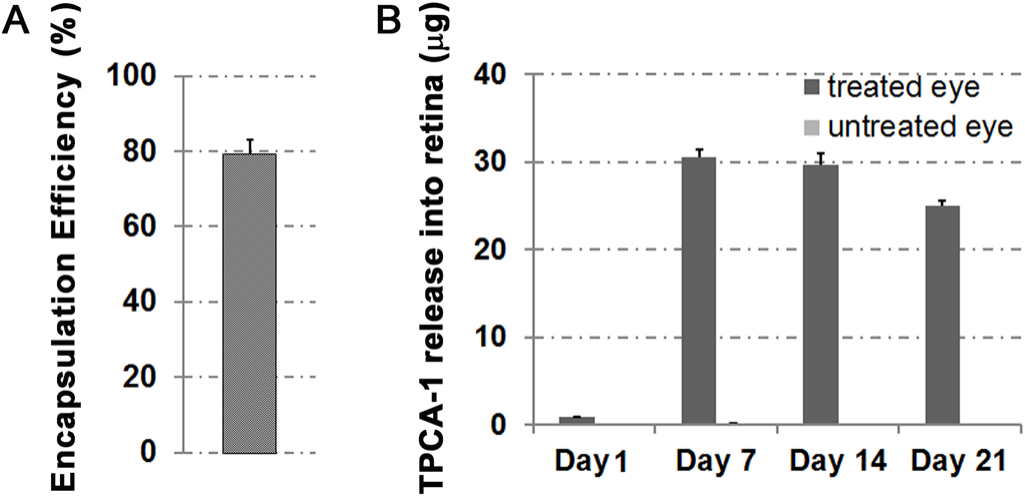
Encapsulation efficiency and *in vivo* drug release profiles. (A) Encapsulation efficiency of TPCA-1 by a solvent-evaporation process is around 80%. (B) Choroid/RPE/retina TPCA-1 release profile at 1, 7, 14, and 21 days post retrobulbar administration of TPCA-1/PLGA (0.8 mg TPCA-1/10 mg PLGA in 100 μL PBS) microparticles. Data expressed as mean±SD, n = 3 (individual experimental mice).

The encapsulation efficiency of TPCA-1 into microspheres was calculated using the difference between two different phases: the amount of TPCA-1 added to the microsphere-forming solution and the amount free in the external phase after microspheres had formed. The microsphere suspension was centrifuged for 10 min at 15,000 rpm, and the supernatant was analyzed for non-entrapped (i.e., free) TPCA-1 by HPLC with UV detection at 310 nm. Encapsulation was further confirmed by directly checking the amount of TPCA-1 loaded into microspheres by dissolving the polymer complexes in 100% ethanol. Our preliminary study shows that TPCA-1 encapsulation efficiency was approximately 80% (Fig. 2A) under the conditions outlined in Fig. 1A.

### In vivo release and evaluation of distribution, safety, and inflammatory response to microspheres

To evaluate TPCA-1 release *in vivo*, TPCA-1-loaded PLGA (10 mg of microparticles loaded with 0.8 mg TPCA-1 suspended in 100 μL PBS) microparticles were unilaterally retrobulbar-injected into C57B/L6 mouse eyes using a 30-gauge needle. Mice were euthanized at 1, 7, 14, or 21 days after injection, and the TPCA-1 level in the treated and untreated homogenized retinal lysate was determined by HPLC after extraction with dichloromethane. A constant release of TPCA-1 was measured in the retinas as late as 21 days after TPCA-1-loaded PLGA retrobulbar injection (Fig. 2B), indicating that TPCA-1 can directly diffuse across the sclera to reach the posterior segment of the eye, including the retina. Interestingly, no significant level of TPCA-1 was detected in the untreated contralateral eye, suggesting that the released drug was confined to the treated eye.

### TPCA-1-loaded PLGA microparticle injection is safe to retinal tissue and the immune system

**Fig 3:**
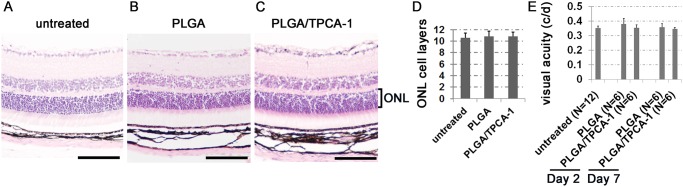
Assessment of retinal histology and visual function. (A-C) H&E-stained images of the retinas from untreated mice (A), mice injected with sham-loaded PLGA microparticles (10 mg PLGA in 100 μL PBS) (B), and mice injected with TPCA-1-loaded PLGA (PLGA/TPCA-1) microparticles (0.8 mg TPCA-1/10 mg PLGA in 100 μL PBS) (C). The tissues were collected at 7 days after treatment. (D) The quantitative comparison of the cell layers in the outer nuclear layer (ONL) among untreated mice, mice treated with PLGA or PLGA/TPCA-1 at 7 days post treatment. Data expressed as mean±SD, n = 3 (individual experimental mice). (E) Visual acuity measured by OKR from eyes treated with PLGA or PLGA/TPCA-1 in comparison with untreated control eyes. Data expressed as mean±SD. N = the number of eyes tested.

To determine the possible cellular toxicity of TPCA-1-loaded PLGA, various organs were excised from the euthanized mice pretreated with TPCA-1-loaded PLGA, PLGA alone, or without any treatment. H&E staining of treated and untreated retina revealed no evidence of retinal detachment or cellular toxicity due to the retrobulbar injection procedure itself or the treatment of TPCA-1-loaded PLGA (Fig. 3A-C). The Outer Nuclear Layer (ONL) consists of 10–11 rows of photoreceptor nuclei in the control and treated mice (Fig. 3D). To test whether mice were visually impaired due to the injection with the PLGA alone or the PLGA loaded with TPCA-1, the spatial frequency and contrast sensitivity thresholds of the optokinetic response (OKR) were measured [38]. The spatial frequency threshold of the OKR averaged 0.352±0.015 cycles per degree (c/d) for untreated mice; and 0.380±0.038 c/d or 0.359±0.024 c/d at 2 or 7 days after the treatment for the PLGA-treated mice, respectively. The OKR threshold for the mice treated with TPCA-1-loaded PLGA averaged 0.355±0.021 c/d and 0.347±0.009 c/d after 2 and 7 days of the treatment. These OKR spatial frequency thresholds in the TPCA-1-loaded PLGA-treated mice showed no significant difference compared with untreated or PLGA-treated mice, suggesting normal visual acuity in the TPCA-1-loaded PLGA-treated mice (Fig. 3E).

**Fig 4:**
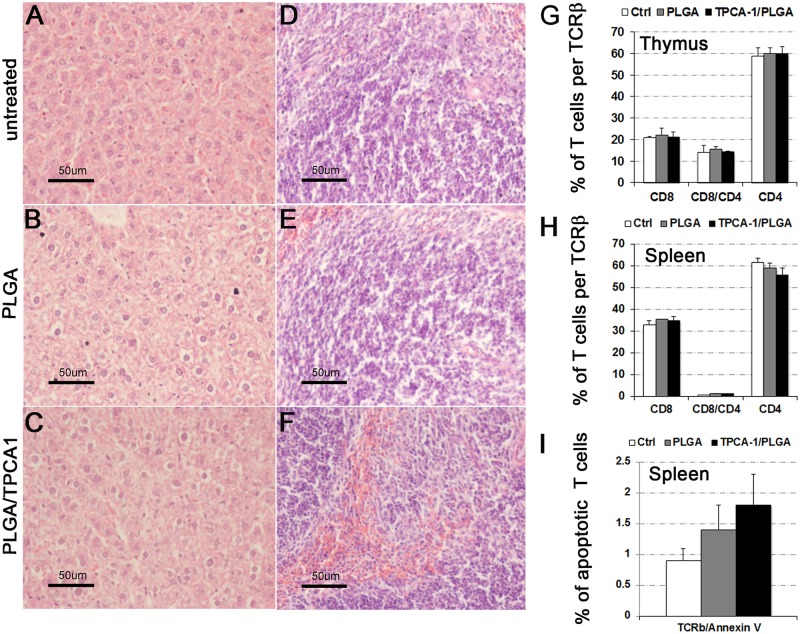
Histological and flow cytometric analyses of liver, spleen, and T cell populations. (A) H&E-stained sections of liver (A, C, and E) and spleen (B, D, and F) tissues on day 7 after PLGA (10 mg PLGA in 100 μL PBS) (A and B) or PLGA/TPCA-1 (0.8 mg TPCA-1/10 mg PLGA in 100 μL PBS) (C and D) retrobulbar injection. The eyes without any injection were used for control (E and F). (G-I) Flow cytometry analysis of CD4^+^ and CD8^+^ single-positive and CD4^+^/CD8^+^ double-positive T cell populations in thymus (G) and spleen (H). Apoptotic assessment of the TCR^+^ lymphocytes was performed on splenocytes using flow cytometry for annexin V exposure on the cell surface (I). Data expressed as mean±SD, n = 3 for each group.

The TPCA-1 administered systemically was reported to cause cellular toxicity in the liver and immune system [13, 29, 30]. To this end, we asked whether sustained release of TPCA-1 from retrobulbar-administered TPCA-1-loaded PLGA microparticles would cause any toxicity to these tissues. Histology analysis of liver and spleen showed no significant difference in the treated and control mice, and no necrosis was observed (Fig. 4A-F). Next, we studied the lymphocytes and monocytes in lymphoid tissues at 7 days after TPCA-1/PLGA treatment using FACS analysis. A single retrobulbar injection of TPCA-1/PLGA (0.8 mg TPCA-1/10 mg PLGA in 100 μL PBS) microparticles did not alter the proportion of CD4^+^/CD8^+^ double-positive and CD4^+^ or CD8^+^ single-positive T cells in either thymus or spleen (Fig. 4G, H). To assess the drug toxicity necessary to cause cell death in T cells, the TCR-β-positive cells were stained with annexin V and showed no significant increase in cell death (Fig. 4I). Thus, low-dose retrobulbar administration of the IKK2 inhibitor TPCA-1 in PLGA microparticles did not cause systemic toxicity to the immune system. This is consistent with our data showing that a significant level of TPCA-1 was detected only in the eye injected with TPCA-1-loaded PLGA and not in the contralateral eye, indicating that the systemic level of TPCA-1 was very low.

### TPCA-1-loaded PLGA microparticles inhibit laser-induced choroid neovascularization

**Fig 5:**
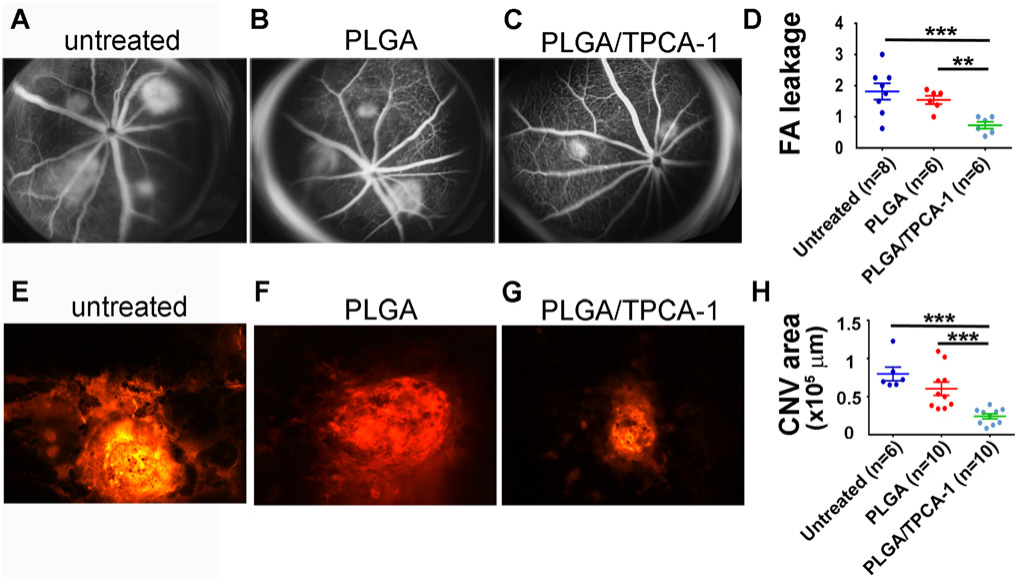
Effects of retrobulbar-injected TPCA-1-loaded PLGA microparticles on laser-induced CNV formation. (A-C) Representative images of fluorescein angiogram were taken from the eyes receiving no injection (A), PLGA (10 mg PLGA in 100 μL PBS) injection (B), and PLGA/TPCA-1 (0.8 mg TPCA-1/10 mg PLGA in 100 μL PBS) injection (C) at 7 days post drug treatment and laser injury. Laser burn was performed in the eyes immediately after retrobulbar injection. (D) Quantification of the fluorescein-leakage score was measured as described in Materials and Methods. (E-G) Representative images of laser-induced CNV stained by isolectin B4. The eyes were injected with PLGA (F) or PLGA/TPCA-1 (G) in comparison with eyes receiving no injection (E). The images were taken and calculated at 7 days after laser injury. (H) Area of CNV in each eye under the indicated conditions, measured from the isolectin B4 staining. The images in E-G were taken and calculated at 7 days after laser injury. “Untreated” represents the results from the eyes without any injection. Data is expressed as mean±SEM.; n = the number of mice used for each condition. The statistical analysis performed for all comparisons was one-way anova followed by *post hoc* Bonferroni *t*-tests using ProStat Ver5.5. **p < 0.01, ***p < 0.005.

To determine the effect of TPCA-1-loaded PLGA on the laser-induced CNV *in vivo*, we retrobulbarly injected the mice with 10 mg of microspheres pre-loaded with 0.8 mg TPCA-1 and suspended in 100 μL PBS or with sham-loaded with PLGA microspheres, as described in Material and Methods. The fundus photographs of the mice that had been grouped as untreated, treated with PLGA, or treated with TPCA-1-loaded PLGA were taken at 7 days after laser injury. A representative photograph from each group is shown in the Fig. 5. While most of the uninjected and PLGA-injected mice developed pathologically significant leakages (grade 1.812±0.563 for un-injected mice; 1.542±0.25 for PLGA-injected mice), the mice injected with the TPCA-1-loaded PLGA developed significantly smaller leakages (0.729±0.229) in the same period (P<0.001, Fig. 5D). The retrobulbar administration of TPCA-1-loaded PLGA reduced the leakage size by two fold (Fig. 5D). To further confirm this observation, isolectin-4B staining was performed to visualize the neovasculature of the CNV areas. Consistent with reduced leakage, CNV size was significantly smaller in the animals treated with the TPCA-1-loaded PLGA, compared with mice without injection or injection with sham-loaded PLGA (Fig. 5H). This data clearly indicates that TPCA-1-loaded PLGA has great potential to restrict CNV growth *in vivo*.

### Treatment with the TPCA-1-loaded PLGA inhibits macrophage recruitment into laser lesions

**Fig 6:**
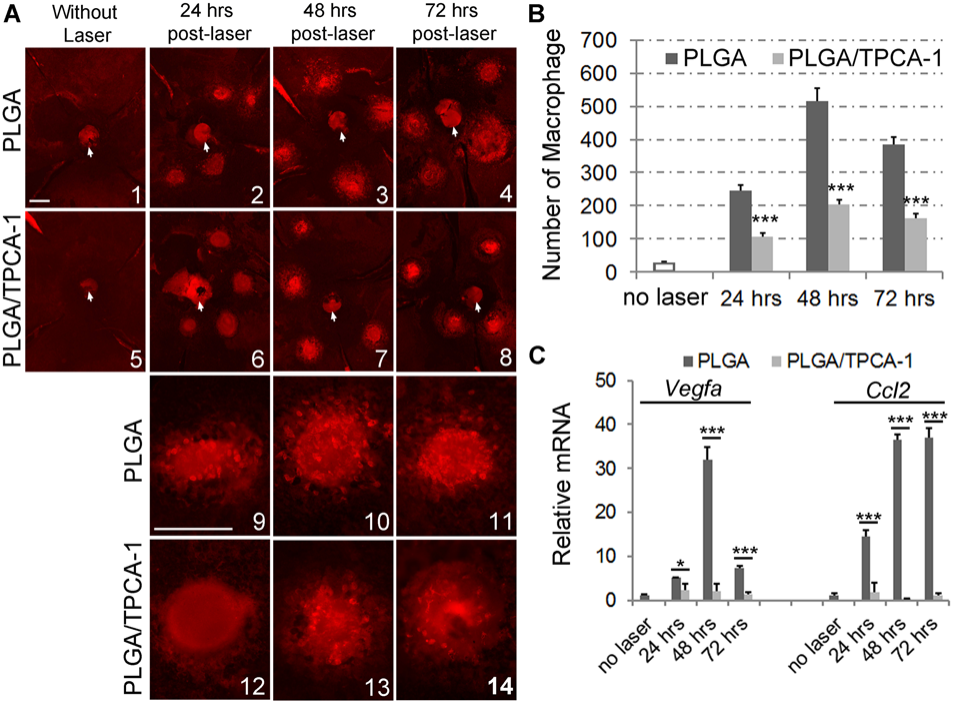
Effects of TPCA-1-loaded PLGA microparticles on macrophage recruitment to the laser lesions. Retrobulbar injection of 100-μL microparticle suspensions containing either PLGA (10 mg) alone or PLGA loaded with TPCA-1 (10 mg/0.8 mg) was performed on the mice at age of 8-week-old, and followed immediately by laser burn of Bruch’s membrane. After 24, 48, or 72 hours, mice were euthanized, and the eyes were dissected and fixed in 4% PFA. The posterior eye cups with attached RPE layer were washed with 1xPBS, stained with F4/80 antibody, and flat-mounted for fluorescent imaging. (A) The representative flat-mount images show that F4/80-stained macrophages appear to have migrated to CNV lesions at 24, 48, or 72 hrs post-laser. Images 1–8 of the laser lesion area on the whole-mounted eye cups were taken at low magnification, and the images 9–14 were taken at higher-magnification. The white arrows indicate optic disks. The scale bar in image A1 represents the magnification of images A1–8, and the scale bar in A9 represents the magnification of A9–14. Scale bar, 250 μm. (B) The number of anti-F4/80-stained macrophages in the choroid/RPE surface were counted and expressed as mean±SD, n = 4 eyes for each condition. Statistics were performed using a two-tailed Student’s *t*-test to compare the values between the PLGA and PLGA/TPCA-1-treated eyes. ***p < 0.005. (C) mRNA expression of *Vegfa* and *Ccl2* determined via qPCR. RNA samples were collected from the choroid/RPE tissues of control and treated mice. The CT method normalized to *Gapdh* was used to analyze relative changes in gene expression. The relative expression (arbitrary units) is expressed as a ratio. Data are expressed as mean±SD, n = 3 experiments. Statistics were performed using a two-tailed Student’s *t*-test to compare the values between the PLGA- and PLGA/TPCA-1- treated eyes. *p < 0.05, ***p < 0.005.

NF-κB transcription factors regulate inflammatory processes by controlling the expression of proinflammatory cytokines and chemokines, many of which are potent macrophage chemoattractants. Macrophage recruitment to choroids contributes to laser-induced CNV formation [31]. To test whether IKK2/NF-κB inhibition mediated by TPCA-1-loaded PLGA affects laser-induced macrophage recruitment into the choroid/RPE, laser injury was performed to induce CNV in the mice preinjected with PLGA alone or the PLGA loaded with TPCA-1. Anti-F4/80-stained macrophages were counted from the flat-mounted choroid/RPE surface collected at 24, 48, and 72 hours after laser treatment (Fig. 6A, B). Without laser injury, a minimal number of macrophages were detected (Fig. 6A, B). Laser injury induced significant trafficking and accumulation of macrophages in the lesions, peaking at 48 hrs in untreated and the PLGA-treated mice compared with the uninjured retina (Fig. 6A, B). The treatment with theTPCA-1-loaded PLGA significantly reduced the number of macrophages in the retina at 24, 48, and 72 hrs after laser treatment (Fig. 6A, B). We conclude that treatment with the TPCA-1-loaded PLGA reduces laser-induced macrophage recruitment into the laser lesions. Furthermore, we examined the expression of *Vegfa* and *Ccl2* that were shown to be chemotactic for macrophage and regulated by NF-κB signaling [2, 32–35]. Consistent with reduced macrophage recruitment, treatment with TPCA-1 loaded PLGA polymer significantly inhibited the laser-induced expressions of *Vegfa* and *Ccl2* (Fig. 6D).

## Discussion

AMD is the leading cause of blindness among adults over the ages of 60 and affects as many as 15 million of Americans. Although neovascular (wet) AMD contributes to only 10% of all AMD afflictions, it accounts for 90% of the severe vision loss caused by AMD. The current treatment for wet AMD is blocking VEGF activity in the eye to reduce CNV leakage and growth. However, a majority of patients are unresponsive to the treatment, and CNV may progress even during the treatment [36–38]. Developing a therapeutic strategy that targets multiple angiogenesis pathways other than just VEGF may provide a potentially more powerful medication treatment. The NF-kB pathway is a key player in regulating hypoxia and chronic inflammation, which is well recognized as an early event leading to CNV pathogenesis and progression [3, 39]. Intravitreal or retrobulbar injection of the IKK2/NF-κB inhibitor TPCA-1 suppresses CNV formation in the laser-induced CNV mouse model [8]. The effects are mediated by the targeting of multiple angiogenic factors, including VEGF [8].

As a chronic retinal disease with a slow, progressive pathogenic process leading to choroid neovascularization and consequent blindness, AMD requires long-term treatment with anti-angiogenic molecules. The development of a drug delivery system that reduces the frequency of operational intervention and drug dose is in demand. Microparticles provide an alternative to frequent intravitreal or periocular injection, as they release the drug sustainably for several weeks or months.

In the present study, the potential for sustained release of TPCA-1 into retina using PLGA microparticles as therapeutic drug carriers in the treatment of laser-induced CNV was investigated. The biodegradable PLGA microparticles gradually degrade and release the packed drug until degradation is completed. Microparticles offer higher drug loading and a longer time for drug release than nanoparticles, which reduces administration frequency and makes microparticle a better option. In general, the higher the homogenization speed and/or the ratio of internal PLGA to polyvinyl alcohol are used, the smaller the microsphere is generated. By adjusting the PLGA molecular weight and polymer size, high encapsulation efficiency, low initial release, and sustained second-phase release (more than 1 month) can be achieved. This result suggests that small amount of TPCA-1 is progressively released from the carrier microparticles. Importantly, our results also show that TPCA-1 reaches the subretinal space after it is released from particles injected into the retrobulbar space. This indicates that TPCA-1-loaded PLGA can be injected periocularly, which will reduce the risks associated with intraocular injection. TPCA-1/PLGA retrobulbar treatment not only resulted in a significant reduction of laser-induced CNV formation, but also effectively halted the recruitment of macrophages into laser lesions at 24, 48, and 72 hours post laser injury.

Safety is a major concern when tampering with NF-κB signaling, as this pathway plays key roles in many biological processes, particularly in innate immunity and cell survival [13–16]. The genetic and pharmacologic inhibition of NF-κB in eye was previously shown to be safe without significant toxicity to the eye [8]. In the present study, a significant level of TPCA-1 was only detected in the injected eye, but not in the contralateral eye, suggesting that a minimal amount of TPCA-1 was released into the systemic circulation. In this regard, we believe that local administration of IKK2 inhibitors into the eye will generate ocular therapeutic effects, without causing noticeable toxicity at the systemic level. Systemic usage of TPCA-1 was demonstrated to reduce T lymphocyte population [30], which was not the case in our intraocular injection of the drug. This is consistent with our previous observation that lack of *Ikk2* in the retina does not cause detrimental effects to retinal development and vision function [8]. RPE cells are not sensitive to TNF-α-induced cell death, even in the presence of IKK2 inhibition [8]. Microparticles themselves do not generate any noticeable side effects to the eye histology and visual function.

In summary, the IKK2 inhibitor TPCA-1 can be slowly released into the posterior segment of the eye from microparticle carriers injected into the retrobulbar space and prevent laser-induced CNV formation, which indicates its potential for treatment of wet AMD and other retinal vascular disorders using this inhibitor. This finding also suggests that local IKK2 inhibition for treatment of other chronic and genetic angiogenesis diseases should be reconsidered.
